# The most used questionnaires for evaluating telemedicine services

**DOI:** 10.1186/s12911-021-01407-y

**Published:** 2021-02-02

**Authors:** Sadrieh Hajesmaeel-Gohari, Kambiz Bahaadinbeigy

**Affiliations:** 1 Medical Informatics Research Center, Institute for Futures Studies in Health, Kerman University of Medical Sciences, Kerman, Iran; 2 Gastroenterology and Hepatology Research Center, Institute of Basic and Clinical Physiology Sciences, Kerman University of Medical Sciences, Kerman, Iran

**Keywords:** Telemedicine, Evaluation, Questionnaire

## Abstract

**Background:**

Questionnaires are commonly used tools in telemedicine services that can help to evaluate different aspects. Selecting the ideal questionnaire for this purpose may be challenging for researchers. This study aims to review which questionnaires are used to evaluate telemedicine services in the studies, which are most common, and what aspects of telemedicine evaluation do they capture.

**Methods:**

The PubMed database was searched in August 2020 to retrieve articles. Data extracted from the final list of articles included author/year of publication, journal of publication, type of evaluation, and evaluation questionnaire. Data were analyzed using descriptive statistics.

**Results:**

Fifty-three articles were included in this study. The questionnaire was used for evaluating the satisfaction (49%), usability (34%), acceptance (11.5%), and implementation (2%) of telemedicine services. Among telemedicine specific questionnaires, Telehealth Usability Questionnaire (TUQ) (19%), Telemedicine Satisfaction Questionnaire (TSQ) (13%), and Service User Technology Acceptability Questionnaire (SUTAQ) (5.5%), were respectively most frequently used in the collected articles. Other most used questionnaires generally used for evaluating the users’ satisfaction, usability, and acceptance of technology were Client Satisfaction Questionnaire (CSQ) (5.5%), Questionnaire for User Interaction Satisfaction (QUIS) (5.5%), System Usability Scale (SUS) (5.5%), Patient Satisfaction Questionnaire (PSQ) (5.5%), and Technology Acceptance Model (TAM) (3.5%) respectively.

**Conclusion:**

Employing specifically designed questionnaires or designing a new questionnaire with fewer questions and more comprehensiveness in terms of the issues studied provides a better evaluation. Attention to user needs, end-user acceptance, and implementation processes, along with users' satisfaction and usability evaluation, may optimize telemedicine efforts in the future.

## Background

Telemedicine provides healthcare services when patients and healthcare providers are at different locations using Information and Communication Technologies (ICT). The core purpose of telemedicine is to improve the health of individuals and communities by exchanging useful information for various goals, such as preventing diseases, for diagnosis, monitoring, providing treatment, educating healthcare providers, and for conducting research [1, 2].

It is essential to identify the limitations, find approaches to overcome them, and create a reasonable structure to implement and use a telemedicine service successfully. Therefore, precise evaluations of telemedicine services are required [3]. Evaluation is the use of systematic and logical methods to assess the attributes of different aspects of the project, including its design, implementation, operation, and outcome [4]. The telemedicine evaluation process should be done independently and systematically on various features as the feasibility of the project, acceptance by participants, availability of service, technical capabilities of participants, clinical outcomes, user satisfaction, quality, and the cost–benefit of the offered service [3].

There are several methods to evaluate different aspects of a telemedicine project. In order to choose the right evaluation method, several issues should be considered: (1) Considering project goals; (2) determining the required amount of budget, energy, and time for evaluation; (3) selecting the appropriate method based on the predetermined criteria and metrics; (4) using understandable and easy evaluation methods for users; (5) being completely aware of the evaluation method used in the study; and (6) using validated methods for the evaluation process [5].

In the field of telemedicine, the most commonly used tools for evaluating user satisfaction are questionnaires and interviews [6]; and in order to assess the usability of telemedicine systems, usually questionnaires, interviews, observations, self-descriptive, and logging methods are used [7]. The clinical outcomes of telemedicine services are evaluated by means of biometric measurements, quality of life, and disease-specific tools that are all generally questionnaires [8].

Researchers use a variety of questionnaires for various purposes. Some of them are valid questionnaires specified for telemedicine whereas some others are more general. Due to the great variety of questionnaires, it is difficult for researchers to choose the best tool to evaluate their telemedicine services. Therefore, this review aims at listing the most commonly used questionnaires for evaluating telemedicine, which would in turn help researchers to select the most appropriate questionnaire, based on their objectives, to evaluate telemedicine services.

## Methods

### Database and date

This is a scoping review. PubMed database was searched in August 2020 to retrieve articles without date limitation. Approximately, 80–90% of studies conducted in Telemedicine were accessible on PubMed, which sufficed for our purpose; therefore, no other database was used [9].

### Search strategy

The following combinations of keywords were used to find relevant articles in the Title/Abstract search field: *(telemedicine) AND (evaluation OR assessment) AND (questionnaire).*

### Inclusion criteria

Original observational and interventional research articles in which a valid and referenced questionnaire was used to evaluate telemedicine services and systems were included in this study.

### Exclusion criteria

Articles were excluded if they were review articles, non-English language, without full text, not specifically addressing telemedicine, did not have communication between patients and healthcare providers in mHealth, evaluated clinical outcomes, evaluated acceptance of telemedicine prior to implementation, did not include details about the questionnaires used, questionnaires that have not been validated, or combined questionnaires.

### Article selection

First, all retrieved articles were assessed based on title and abstract by one researcher (SH). Next, the same researcher reviewed the full-text of the selected articles. When necessary, the second researcher (K.B), being a telemedicine professional, provided consult. Manual searching was also performed to find additional articles that had used specific evaluation questionnaires to investigate Telemedicine. Finally, a list of all included articles was prepared.

### Data extraction

The following information was extracted from included articles: author, year of publication, journal of publication, evaluation type, and evaluation questionnaire.

### Data analysis

Descriptive statistics (frequency and frequency percent) was used to analyze data.

## Results

**Fig. 1:**
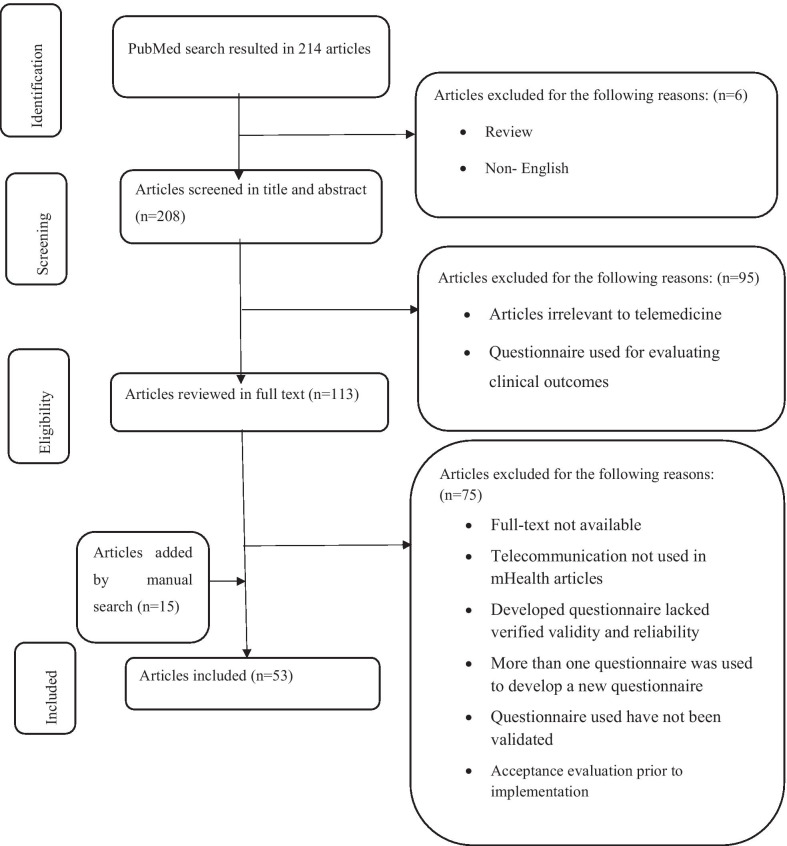
The process of PRISMA in finding and including articles

PubMed database found 214 articles. After removing review articles and those written in a language other than English, 208 articles remained whose titles and abstracts were screened. After excluding 95 articles, the full-text of the 113 remaining articles were reviewed. Finally, 53 articles were included in this study (Fig. 1), from which the required data was extracted (Table 1).

**Table 1:** Information of included articles

Author and year	Journal of publication	Evaluation type	Evaluation questionnaire
Layfield et al. [10] 2020	Head & Neck	Usability	Telehealth Usability Questionnaire (TUQ) [18]
Mostafa et al. [11] 2020	Journal of Dermatological Treatment	Usability	Telehealth Usability Questionnaire (TUQ) [18]
Whitehouse et al. [12] 2020	Research in Gerontological Nursing	Usability	Telehealth Usability Questionnaire (TUQ) [18]
Vaughan et al. [13] 2020	Telemedicine and e-health	Usability	Telehealth Usability Questionnaire (TUQ) [18]
Cheng et al. [14] 2020	JAAOS Global Research & Reviews	Satisfaction, Usability	Telehealth Satisfaction Scale (TeSS) [63] and Telehealth Usability Questionnaire (TUQ) [18]
Mehra et al. [48] 2020	Journal of Medical Internet Research	Usability	Usefulness, Satisfaction, and Ease of use (USE) questionnaire [64]
Lin et al. [20] 2020	Journal of Medical Internet Research	Satisfaction	Modified Telemedicine Satisfaction Questionnaire (TSQ) [26]
Christensen et al. [31] 2020	Telemedicine and e-health	Satisfaction	Client Satisfaction Questionnaire 3 (CSQ 3) [65]
Christensen et al. [30] 2020	Telemedicine and e-health	Satisfaction	Client Satisfaction Questionnaire 8 (CSQ 8) [65]
Leppert et al. [49] 2020	Clinical research in cardiology	Acceptance	Florida Patient Acceptance Survey (FPAS) [66]
McGloin et al. [41]2020	Journal of Medical Internet Research	Satisfaction	Telemedicine Satisfaction and Usefulness Questionnaire (TSUQ) [42]
Talal et al. [21] 2019	Telemedicine and e-health	Satisfaction	Modified Telemedicine Satisfaction Questionnaire (TSQ)[26]
Le et al. [22] 2019	Digestive Diseases and Sciences	Satisfaction	Patient Satisfaction Questionnaire (PSQ) [67] and Telemedicine Satisfaction Questionnaire (TSQ)[26]
Serwe et al. [15] 2018	International Journal of Telerehabilitation	Usability	Telehealth Usability Questionnaire (TUQ) [18]
van der Meij et al. [50] 2018	Journal of Medical Internet Research	Process	Model of Linnan and Steckler [68]
Safdari et al. [33] 2018	Journal of diabetes and metabolic disorders	Usability	Questionnaire for User Interaction Satisfaction (QUIS) [69]
Losiouk et al. [23] 2018	Journal of Telemedicine and Telecare	Satisfaction	Modified Telemedicine Satisfaction Questionnaire (TSQ) [26]
Host et al. [51] 2018	Clinical & experimental optometry	Satisfaction	Modified patient satisfaction with videoconferencing for specialty consultation questionnaire [70]
Hosseini et al. [34] 2018	The open medical informatics journal	Usability	Questionnaire for User Interaction Satisfaction (QUIS) [69]
Hatton et al. [43] 2018	Journal of pharmacy practice	Satisfaction	Modified Patient Assessment of Communication during Telemedicine (PACT) questionnaire [44]
D'Hooghe et al. [52] 2018	Multiple sclerosis and related disorders	Satisfaction	Dutch version of Quebec User Evaluation of Satisfaction with Assistive Technology (D-QUEST 2.0) [71]
Ammenwerth et al. [53] 2018	JMIR cardio	Satisfaction	Delone and McLean Information System Success Model [72]
Torbjørnsen et al. [27] 2018	JMIR human factors	Acceptance	Service User Technology Acceptability Questionnaire (SUTAQ) [29]
van Rosmalen-Nooijens et al. [62] 2017	Journal of Medical Internet Research	Acceptance	Web Evaluation Questionnaire (WEQ) [73]
Segura-Sampedro et al. [24] 2017	Annals of medicine and surgery	Satisfaction	Telemedicine Satisfaction Questionnaire (TSQ) [26]
Oliveira et al. [54] 2017	JMIR Med Education	Satisfaction	Wang’s an e-learning satisfaction model [74]
Agnisarman et al. [46] 2017	Applied ergonomics	Usability, Acceptance	IBM Computer System Usability Questionnaire (CSUQ) [75] and the NASA Task Load Index test [76] and Modified Technology Acceptance Model (TAM) [77]
Serwe et al. [16] 2017	International Journal of Telerehabilitation	Usability	Telehealth Usability Questionnaire (TUQ) [18]
Yu et al. [17] 2017	Disability and Rehabilitation: Assistive Technology	Usability	Telehealth Usability Questionnaire (TUQ) [18]
Parmanto et al. [18] 2016	International Journal of Telerehabilitation	Usability	Telehealth Usability Questionnaire (TUQ) [18]
Smaradottir et al. [36] 2016	Journal of Telemedicine and Telecare	Usability	System Usability Scale (SUS) questionnaire [78]
Fields et al. [32] 2016	Sleep	Satisfaction	Client Satisfaction Questionnaire 8 (CSQ 8) [65]
Dario et al. [28] 2016	International journal of integrated care	Acceptance	Service User Technology Acceptability Questionnaire (SUTAQ) [29]
Hirani et al. [29] 2016	Journal of Telemedicine and Telecare	Acceptance	Service User Technology Acceptability Questionnaire (SUTAQ) [29]
Alanzi et al. [35] 2016	JMIR research protocols	Usability	Questionnaire for User Interaction Satisfaction (QUIS) [69]
Roberts et al. [55] 2015	The Australian journal of rural health	Satisfaction	The questionnaire based on a validated instrument used in teledermatology [79]
Poulsen et al. [56] 2015	International journal of rheumatic diseases	Satisfaction	Questionnaire used to evaluate a similar medical oncology telemedicine service [80]
Ligons et al. [37] 2014	International journal of medical informatics	Usability	System Usability Scale (SUS) questionnaire [78]
Lacerda et al. [38] 2014	Journal of biomedical informatics	Usability	System Usability Scale (SUS) questionnaire [78]
Vélez et al. [57] 2014	Journal of midwifery & women's health	Usability	Health-Information Technology Usability Survey (Health-ITUES) [81]
Penteado et al. [58] 2014	JMIR medical informatics	Satisfaction	Satisfaction with Amplification in Daily Life (SADL) [82]
Kwon et al. [45] 2014	Telemedicine and e-health	Usability	Modified Post Study System Usability Questionnaire (PSSUQ) [83]
McFarland et al. [39] 2013	Telemedicine and e-health	Satisfaction	Modified Ware et al. ‘s Patient Satisfaction Questionnaire (PSQ) [84]
Parra et al. [47] 2012	Interactive journal of medical research	Acceptance	Modified Technology Acceptance Model (TAM) [77]
Schutte et al. [19] 2012	International Journal of Telerehabilitation	Usability	The After- Scenario Questionnaire (ASQ) [85] and The Post-Study System Usability Questionnaire (PSSUQ) [83] and Telehealth Usability Questionnaire (TUQ) [18]
Dechêne et al. [25] 2011	International Journal of Telerehabilitation	Satisfaction	Telemedicine Satisfaction Questionnaire (TSQ) [26]
Scalvini et al. [59] 2009	Telemedicine and e-health	Satisfaction	NHS patient questionnaire [86]
Agha et al. [44] 2009	Telemedicine and e-health	Satisfaction	Patient Assessment of Communication during Telemedicine (PACT) questionnaire [44]
Bakken et al. [42] 2006	Journal of the American Medical Informatics Association	Satisfaction	Telemedicine Satisfaction and Usefulness Questionnaire (TSUQ) [42]
Kim et al. [60] 2004	Telemedicine and e-health	Satisfaction	Patient attitudes and satisfaction questionnaire [87]
Eminovic et al. [61] 2004	Journal of Medical Internet Research	Satisfaction	Modified Telemedicine Perception Questionnaire (TMPQ) [88]
Yip et al. [26] 2003	Journal of Telemedicine and Telecare	Satisfaction	Telemedicine Satisfaction Questionnaire (TSQ) [26]
Wallace et al. [40] 2002	BMC family practice	Satisfaction	Ware et al. ‘s Patient Satisfaction Questionnaire (PSQ) [84]

### Journal of publication

Nine articles were published in the *Telemedicine and e-health* Journal (17%), six in the *Journal of Medical Internet Research* (11.5%), four in the *International Journal of Telerehabilitation* (7.5%), four in the *Journal of Telemedicine and Telecare* (7.5%), and the remaining articles were published in other journals (n = 30, 56.5%).

### Evaluation type

A questionnaire was used in 26 articles (49%) to evaluate patients’ or physicians’ satisfaction; in 18 articles to evaluate system usability (34%), in 6 articles to evaluate acceptance (11.5%), and in one article to evaluate the implementation process (2%). In 2 articles a questionnaire was used to evaluate the usability and the acceptance or satisfaction of individuals (3.5%).

### Evaluation questionnaire

The final list of articles showed that the Telehealth Usability Questionnaire (TUQ) (n = 10, 19%) [10–19] and Telemedicine Satisfaction Questionnaire (TSQ) (n = 7, 13%) [20–26] were the most commonly used. Then, the Service User Technology Acceptability Questionnaire (SUTAQ) (n = 3, 5.5%) [27–29], the Client Satisfaction Questionnaire (CSQ) (n = 3, 5.5%) [30–32], the Questionnaire for User Interaction Satisfaction (QUIS) (n = 3, 5.5%) [33–35], the System Usability Scale (SUS) questionnaire (n = 3, 5.5%) [36–38], the Patient Satisfaction Questionnaire (PSQ) (n = 3, 5.5%) [22, 39, 40], the Telemedicine Satisfaction and Usefulness Questionnaire (TSUQ) (n = 2, 3.5%) [41, 42], the Patient Assessment of Communication during Telemedicine (PACT) questionnaire (n = 2, 3.5%) [43, 44], the Post Study System Usability Questionnaire (PSSUQ) (n = 2, 3.5%) [19, 45] and the Technology Acceptance Model (TAM) (n = 2, 3.5%) [46, 47] were the most used questionnaires, respectively. The rest of the articles had used other questionnaires (n = 18, 34%) [14, 19, 46, 48–62] and 4 articles had used more than one questionnaire.

### The most used questionnaires

**Fig. 2:**
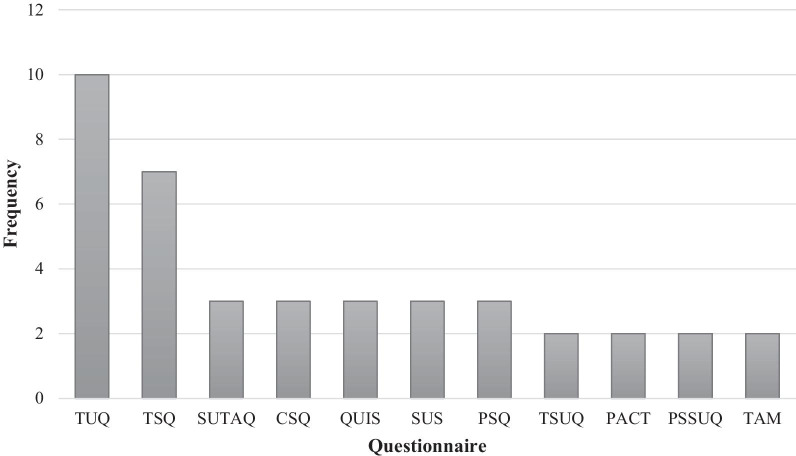
The frequency of the most used questionnaires for evaluating telemedicine services

Out of 59 used questionnaires in the studies, the most frequently (more than two times) used were TUQ, TSQ, SUTAQ, CSQ, QUIS, SUS, PSQ, TSUQ, PACT, PSSUQ, and TAM, which have been shown in Fig. 2.

Some information about these questionnaires are presented below:

Parmanto et al. in 2016, formally introduced TUQ. This questionnaire evaluates the usability of telehealth services. It has 21 items that are based on 6 criteria including usefulness (3 items), ease of use and learnability (3 items), interface quality (4 items), interaction quality (4 items), reliability (3 items), and satisfaction and future use (4 items) [18]. The TUQ was designed using existing telehealth and general usability questionnaires and includes all usability aspects.

The TSQ was developed by Yip et al. in 2003 for evaluating patient satisfaction in using telemedicine. This questionnaire has 14 items with no categories [26].

Hirani et al. presented SUTAQ in 2017, as a tool to judge the acceptability of telehealth services by investigating user opinions. This questionnaire consists of 22 items that is categorized in six sectors: benefits (9 items), privacy (4 items), personal care skill (3 items), substitution (3 items), and satisfaction (3 items) [29].

Bakken et al. in 2006, designed TSUQ. This questionnaire evaluates the satisfaction and usefulness criteria of video consultation and telephone monitoring. This instrument has 26 items in two satisfaction (21 items) and usefulness (5 items) sections [42].

PACT questionnaire was introduced by Agha et al. in 2009. This questionnaire measures the satisfaction of patients and physicians of their communication by asking 33 questions. These questions are asked about the quality of physician and patient communication (16 questions), clinical skills (9 questions), interpersonal skills (6 questions), and comfortable of visit (2 questions) sectors [44].

Attkisson et al. in 1982, firstly introduced CSQ. This questionnaire evaluates the general users’ satisfaction with different services. The original version of CSQ has 18 items; however, the questionnaire has other versions in which there are only 3 or 8 items [65].

QUIS was developed by Chin et al. in 1988. It is a usability testing instrument that has been designed to measure the satisfaction of users about their interaction with the computer interface. QUIS consists of 27 items in five sections. The first section measures the overall satisfaction, and the four others measure user satisfaction regarding screen, terminology and information, learning, and system capabilities aspects [69].

The SUS questionnaire was created by Brooke et al. in 1986 for testing the usability of electronic systems. This tool has ten items with no specific categories [78].

The PSQ was firstly designed by Ware et al. in 1983 for measuring the satisfaction of patients with using medical cares. The original version of this questionnaire has 80 items measured in seven categories, such as overall satisfaction, technical quality, interpersonal mode, communication, financial features, time spent with physician, availability, and convenience [84]. A shorter version of this questionnaire also exists which consists of 18 items [67].

PSSUQ was developed by the International Business Machines Corporation (IBM) to evaluate users’ satisfaction at the end of the study. This questionnaire has three versions. The last version consists of 16 questions with seven Likert scales. These questions are arranged is such way to evaluate matters in the three following sections: system usefulness, information quality, and interface quality [75, 83].

TAM is a model that shows how users use and accept a technology. A questionnaire that has been developed based on this model was presented by Davis in 1989. This questionnaire investigates 12 items that are generally concerned with two matters: the perceived usefulness and perceived ease of use sections with 12 items [77].

## Discussion

The TUQ, TSQ, SUTAQ were the three most common telemedicine-specific questionnaires used in the retrieved articles, respectively. The four most used general questionnaires related to satisfaction and usability evaluation were the CSQ, QUIS, SUS, and PSQ.

In the below sections it will be discussed why each questionnaire is used more frequently. To do this we chose the most three used questionnaires which are designed specific for telemedicine and four general questionnaires that are used in telemedicine evaluation as well.

TUQ is usable for evaluating different types of telehealth systems such as sole target videoconferencing systems, computer and mobile-based systems, and collecting the opinions of both patients and physicians [18]. As TUQ is considerably comprehensive when comparing with other questionnaires such as QUIS and SUS, it is most frequently used for evaluating the usability of telemedicine systems. SUS questionnaire, like QUIS, is a general usability evaluation tool that is also used to evaluate telemedicine systems; yet, unlike QUIS it has no segmentation and it examines fewer items [78]. Also, ever since TUQ has been introduced, SUS has been seldom used.

The TSQ is a preliminary tool for measuring patient satisfaction with telemedicine, and it is used quite frequently. This might be due to the fact that TSQ covers various satisfaction factors such as the quality of care, quality of virtual visits, interpersonal interactions, and also it has fewer number of items [26].

Although TSUQ was also introduced many years ago, it has rarely been used for studies conducted in telemedicine, which may be due to two reasons: (1) it has been designed specifically for telemedicine services provided to diabetes patients, and (2) it investigates more items than other questionnaires especially, TSQ [42].

The CSQ was a generic most used questionnaire for evaluating users’ satisfaction with the telemedicine services. This may be due to the fact that CSQ measures the quality of diverse attributes such as the physical environment, procedure, assistance staff, type of service, treatment staff, amount or length of service, service quality, outcome, and general satisfaction with few items [65]. PSQ is a general questionnaire as well and it is designed to evaluate patient satisfaction. However, with presenting the TSQ, evaluators preferred to use TSQ as a specific questionnaire in assessing the users’ satisfaction of a telemedicine service. Evaluators may use different tools depending on their purpose of investigation, and the only thing that matters is the validity of the used tool.

The SUTAQ is the only questionnaire that specifically designed to gather patients’ opinions about the acceptability of the telehealth systems [29]. While there are various models for assessing technology acceptance, this instrument is used more in this field since it has been specifically designed for evaluating telehealth acceptance.

In terms of evaluation types for telemedicine services, users’ satisfaction, usability, acceptance, and implementation process are non-clinical aspects that have been evaluated by use of validated questionnaires. Approximately, a questionnaire had been used on half of the collected articles for evaluating the satisfaction of telemedicine users; and approximately in a third of the articles, the usability of telemedicine systems was evaluated.

Telemedicine acceptance [27–29, 46, 47, 49, 62] and its implementation process [50] were evaluated in only a few articles. A review study investigating the evaluation methods for telemedicine services in hospitals showed that telemedicine users' satisfaction more frequently evaluated than clinical and economical aspects, and the most commonly used method to evaluate satisfaction was questionnaire. Similar to our result, this study also showed that the development and implementation process of telemedicine had gained less attention [6].

Evaluating the implementation process could be an essential stage for the successful usage of telemedicine services due to showing obstacles and facilitators of implementation [50]. Also, considering the needs of the users and planning the process based on these needs may affect the successful implementation of telemedicine services and increase the rate of their acceptance. Therefore, we recommend that researchers pay more attention to this aspect in the evaluation of telemedicine services. Another study that reviewed the usability evaluation methods of eHealth services for patients who had HIV revealed that questionnaire was the most employed method for evaluation [89].

Based on the researchers’ knowledge, this is the first review study that has identified validated and the most used questionnaires in evaluating telemedicine services. Nevertheless, this study has its own limitations. Only PubMed database was used for searching and retrieving articles. In the search strategy, we used telemedicine as a MeSH term and evaluation and assessment keywords that may use in different grammatical style. Moreover, we restricted our search to the Title/Abstract field. These issues may cause some articles missed from our study. For this reason, we conducted a manual search using the name of telemedicine specific questionnaires in the PubMed database and added additional articles.

## Conclusion

Many questionnaires were used to assess telemedicine services. Some of them were specifically designed to evaluate telemedicine services while others were more general. As the results of this study showed, telemedicine service evaluators should use questionnaires specifically designed for telemedicine to assess its various aspects. However, if an evaluator wants to design a questionnaire for evaluating a telemedicine service, it is better to pay attention to goal-based design, the number of questions, and comprehensiveness in terms of the issues studied.

Users' satisfaction with telemedicine services and the usability of the system have been two of the most frequently investigated issues in telemedicine when comparing with other existing issues in the field. Attention to user needs, end-user acceptance, and implementation processes may optimize telemedicine efforts in the future.

## Data Availability

Not applicable.

## Abbreviations

ICT: Information and communication technologies
TUQ: Telehealth usability questionnaire
TeSS: Telehealth satisfaction scale
USE: Usefulness, satisfaction, and ease of use
TSQ: Telemedicine satisfaction questionnaire
CSQ: Client satisfaction questionnaire
FPAS: Florida patient acceptance survey
TSUQ: Telemedicine satisfaction and usefulness questionnaire
PSQ: Patient satisfaction questionnaire
QUIS: Questionnaire for user interaction satisfaction
PACT: Patient assessment of communication during telemedicine
D-QUEST: Dutch version of Quebec user evaluation of satisfaction with assistive technology
SUTAQ: Service user technology acceptability questionnaire
WEQ: Web evaluation questionnaire
CSUQ: Computer system usability questionnaire
TAM: Technology acceptance model
SUS: System usability scale
Health-ITUES: Health-information technology usability survey
SADL: Satisfaction with amplification in daily life
PSSUQ: Post study system usability questionnaire
ASQ: The after-scenario questionnaire
TMPQ: Telemedicine perception questionnaire
